# Dietary Intake of Carotenoids and Risk of Depressive Symptoms: A Systematic Review and Meta-Analysis

**DOI:** 10.3390/antiox11112205

**Published:** 2022-11-07

**Authors:** Qiong Yu, Fengyu Xue, Zhijun Li, Xinwei Li, Lizhe Ai, Mengdi Jin, Mengtong Xie, Yaqin Yu

**Affiliations:** 1 Faculty of Health, Zhuhai College of Science and Technology, Zhuhai 519000, China; 2 Department of Epidemiology and Biostatistics, School of Public Health, Jilin University, Changchun 130012, China

**Keywords:** depression, carotenoids, oxidative stress, antioxidants, meta-analysis

## Abstract

Given the important role of oxidative stress in the pathogenesis of depression, the potential role of dietary antioxidant supplementation in the prevention of depression has attracted considerable attention. Most studies suggest that dietary carotenoids may play a role in maintaining depressive symptoms due to their antioxidant activity, but some studies concluded the contrary. This study conducted a meta-analysis of observational studies to test the relationship between carotenoid supplements and depressive symptoms. After a comprehensive search of the Cochrane Library, PubMed, Embase Scopus, and Web of Science databases from their inception to 28 July 2022, 12 publications met the inclusion and exclusion criteria, of which 8 were cross-sectional studies, 3 were case–control studies, and 1 was a cohort study, involving a total of 33,466 participants. Pooled meta-analysis found that intake of total carotenoids (OR = 0.61, 95% CI [0.53, 0.71], *p* < 0.01), beta-carotene (OR = 0.61, 95% CI [0.52, 0.70], *p* < 0.01), alpha-carotene (OR = 0.71, 95% CI [0.60, 0.83], *p* < 0.01), lycopene (OR = 0.71, 95% CI [0.55, 0.90], *p* < 0.01), lutein, and/or corn xanthin (OR = 0.53, 95% CI [0.43, 0.66], *p* < 0.01) was significantly inversely associated with depressive symptoms, while beta-cryptoxanthin (OR = 1.07, 95% CI [0.52, 2.21], *p* = 0.86) had no significance. At the same time, this meta-analysis was free of publication bias and heterogeneity. Although further studies are needed to elucidate the causal relationship between carotenoids and depressive symptoms, and to further reveal the mechanism of their association, the results of our meta-analysis suggest that carotenoids are protective factors for depressive symptoms, and dietary intake may help in reducing the risk of depressive symptoms.

## 1. Introduction

Depression is a major public health issue that brings a huge disease burden on individuals, families, and societies all over the world [1]. Depression is a common and serious mental illness characterized by high emotional distress and dysfunction [2]. The main symptoms include depression and lack of pleasure (loss of interest in daily activities) [3], which are often accompanied by headaches, dizziness, and additional symptoms of various cerebrovascular diseases [4]. Studies showed that people with depressive symptoms are currently estimated to account for 4.4% of the world, or about 322 million. In the past 10 years alone, the global number of people suffering from depressive symptoms increased by 18.4% [5]. Depression is expected to become more globally prevalent, with a steady and sustained increase in the number of affected people [5]. Despite extensive research, its pathogenesis has not been fully clarified [6], and its exact cause is still unclear. The pathogenesis and complexity of depression are not only related to genetic components, but also to psychological, environmental, and biological factors, including social and psychological stressors to which a person was exposed in their early years or adulthood [7,8].

Oxidative stress is widely considered to be the key factor in the pathogenesis and pathology of depression, and the brain is more susceptible to an increase in reactive oxygen species than other organs of the body are because it is rich in oxidizable lipids and is the main consumer of oxygen. The imbalance between active oxygen and antioxidant defense can lead to brain dysfunction and abnormal nerve signals [9,10,11,12]. Depression is associated with reduced plasma concentrations of an important antioxidant, vitamin E, which protects the brain from reactive oxygen species-induced damage [13]. Coenzyme Q 10 (CoQ 10) can resist mitochondrial damage caused by oxidation and nitrosation [14], and another antioxidant, N-acetylcysteine (NAC), is effective in reducing hydroxyl free radicals [15]. Dietary supplements such as micronutrients, vitamins, n-3 polyunsaturated fatty acids, and antioxidants can also relieve symptoms and slow down the progression of depression [16]. A clinical trial for depressed patients showed that zinc supplementation could reduce depressive symptoms compared with antidepressants alone [17]. In addition, a cross-sectional study showed that a higher dietary intake of B vitamins (especially biotin) was effective in reducing the incidence of depression, anxiety, and stress among Iranians aged 20–70 [18]. Furthermore, a randomized controlled trial showed that the vitamin group could enhance the response to antidepressant treatment within one year, and advocated the adjunctive use of vitamin B as a safe and inexpensive strategy for managing major depressive disorder in older adults [19]. All these findings indicate that various dietary antioxidants may slow down the development of depressive symptoms. In many different types of antioxidant supplements, carotenoids are antioxidants that help in fighting depression.

Carotenoids are important dietary nutrients with antioxidant effects that widely exist in dark green, yellow, or red vegetables and fruits, such as carrots, tomatoes, and cantaloupes [20]. According to their chemical composition and structure, carotenoids can be divided into two categories: carotene and lutein [21]. More than 700 types of carotenoids have been identified in nature, but the content of 6 kinds of total carotenoids in human serum is relatively high, namely, alpha-carotene, beta-carotene, beta-cryptoxanthin, lycopene, zeaxanthin, and lutein [22]. Studies repeatedly proved that carotenoids in the diet are very effective physical quenchers of singlet oxygen and scavengers of other active oxygen, being able to scavenge free radicals and effectively prevent depression, thus reducing oxidative stress and preventing brain damage [23]. Recent studies showed that carotenoids are candidates for the prevention and treatment of depression due to their antioxidant properties and because they have the advantage of not having associated side effects compared to traditional antidepressants [24,25].

The research on the potential of carotenoid supplementation to maintain or even improve depressive symptoms is still inconsistent. To the best of our knowledge, no published meta-analyses provide quantitative measures of the association between carotenoids and depressive symptoms. Therefore, we performed meta-analysis and a systematic review of published epidemiological studies to quantitatively assess whether carotenoids have any effect on patients with depressive symptoms.

## 2. Materials and Methods

This study was designed and conducted according to Preferred Reporting Items for Systematic Reviews and Meta-Analyses (PRISMA) [26].

### 2.1. Search Strategy

The data search was completed on 28 July 2022. Studies were identified from systematically searching databases Embase, PubMed, Web of Science, Scopus, and Cochrane Library, and supplemented by searching the major relevant review papers and the reference lists from all included studies, which was restricted to the English literature. For a more comprehensive and systematic literature search, we used a combination of controlled vocabulary and free-text terms. The MeSH terms and keywords used for searching were as follows: “α-carotene”, “alpha-carotene”, “beta-carotene”, “β-carotene”, “β-cryptoxanthin”, “beta-cryptoxanthin”, “lutein”, “zeaxanthin”, “lycopene”, “astaxanthin”, “carotene”, “carotenoids”, “depression”, “depressive symptom”, “emotional depression”.

### 2.2. Inclusion and Exclusion Criteria

**Figure 1:**
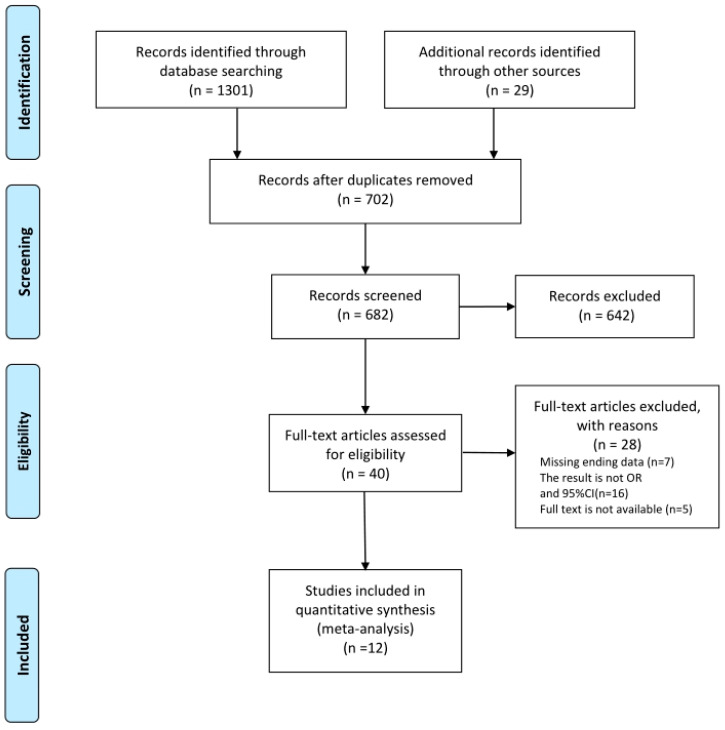
Selection of studies for inclusion in the present meta-analysis.

Two researchers (F.X. and Z.L.) independently screened the literature according to pre-established inclusion and exclusion criteria. Studies that fulfilled the following criteria were eligible: (1) subject with depressive symptoms must have clear judgment criteria; (2) the carotenoid concentration was measured via serum carotenoid concentration; (3) results must include quantitative data of odds ratios (OR) and their 95% confidence interval (95% CI) to assess the risk of carotenoid deficiency between subjects with depressive symptoms and normal subjects. Articles were excluded from the review for the following reasons: (1) research on evaluating the effects of carotenoids on neurological diseases other than depressive symptoms, such as schizophrenia, bipolar disorder, and cognitive impairment; (2) comprehensive studies involving the intake of dietary carotenoids and other antioxidant supplements; (3) studies using nonprimary data such as conference papers, reviews, meta-analyses, and book chapters; (4) research that had not been published in English; (5) in vitro experiments or animal model study. The flowchart in Figure 1 describes the process of research selection in detail.

### 2.3. Selection Process and Data Extraction

First, two researchers (F.X. and Z.L.) independently screened the titles and abstracts of all documents to determine the documents that met the qualification criteria. Then, the same researchers independently searched and evaluated the full texts of the remaining literature. Lastly, the researchers extracted data from eligible studies using standardized data extraction forms, including basic information such as first-author name, year of publication, country, mean age or age range of participants, study design, sample size, depression symptom evaluation method, type of carotenoid, odds ratio (OR), and 95% confidence intervals (95% CI) of the study conclusion; any disagreement was resolved with negotiation and reaching a consensus.

### 2.4. Quality Evaluation

Two investigators (F.X. and Z.L.) independently evaluated the quality and bias risk of each included study using the Joanna Briggs Institute reviewers’ manual (JBI) [27]. There are 8 items in the table, and each item has a score ranging from 0 to 2 points: the determination of inclusion criteria, the description of research subjects, the measurement of exposure, the determination of disease criteria, the identification of confounding factors, confirmation, treatment of confounders, measurement of outcomes, and appropriate statistical analysis. The best article quality is 16 points, and the lowest quality is 0 points. More details can be found in Table 1.

### 2.5. Statistical Analysis

This meta-analysis assesses the association between dietary carotenoid intake and depressive symptoms. As measures of effectiveness, we calculated the odds ratio (OR) 95% and confidence interval (CI). Cochran’s Q statistic and the *I*^2^ statistic were used to evaluate the statistical heterogeneity (defined as significant when *p* < 0.05 or *I*^2^ > 50%). The fixed-effect model was used when there was insignificant heterogeneity. Otherwise, a random-effect model was applied [28]. Publication bias was assessed with the visual inspection of funnel plots and Egger’s test; when the present was corrected, it was assessed via trim and fill analysis [29,30]. All statistical analyses were performed by using R 4.1.2.

## 3. Results

### 3.1. Study Selection

In a database search, we found 1330 articles in the Scopus, PubMed, Embase, Web of Science, and Cochrane Library. First, 682 articles were screened and evaluated through the publication title and abstract. Second, 642 irrelevant publications were discarded according to the predetermined inclusion and exclusion criteria. Therefore, a total of 40 studies were comprehensively reviewed, and 28 of them were excluded. The reasons are as follows: missing ending data (*n* = 7), the result was not OR and 95% CI (*n* = 16), and the full text was not available (*n* = 5). Lastly, the systematic review and meta-analysis comprised 12 observational studies [1,31,32,33,34,35,36,37,38,39,40,41,42]. The flowchart of the literature search including the exclusion criteria is shown in Figure 1.

### 3.2. Study Characteristics

The baseline characteristics of the 12 included articles are summarized in Table 1. Among the 12 studies analyzing the relationship between carotenoids and depressive symptoms, 1 study was performed in the United States [32], 4 studies were performed in China [35,37,39,42], 4 studies were performed in Iran [36,38,40,41], 1 study was performed in Korea [1], 1 study was performed in Italy [1], and 1 study was performed in Japan [34]. All studies were predominantly concentrated on adult populations, including one longitudinal cohort study [1], three case–control studies [33,36,41], and eight cross-sectional studies [32,34,35,37,38,39,40,42]. Five studies were conducted with women [33,36,37,38,40], and the remaining studies were gender-neutral [1,32,34,35,39,41,42]. The publications reported at least one dietary intake of carotenoid level, providing information on the following carotenoids. Thirteen studies met the inclusion and exclusion criteria, of which five, seven, five, three, four, and three studies reported the effects of carotenoids, alpha-carotene, beta-carotene, beta-cryptoxanthin, lutein, and zeaxanthin, respectively.

Of the 12 studies of dietary intakes of carotenoids and the risk of depressive symptoms, 8 studies were associated with reduced levels of depressive symptoms [1,32,33,35,36,37,38,40]. However, Honghan Ge et al. found that there was no simple linear relationship between dietary carotenoid (beta-carotene, lutein, and zeaxanthin) intake and the risk of depressive symptoms, but a U-shaped dose–response relation [39]. In addition, Song Lin et al. found that there was no correlation between the dietary intake of alpha-carotene, beta-carotene, lycopene, and lutein/zeaxanthin, and depressive symptoms. However, when beta-cryptoxanthin intake reached more than 110 ug/1000 Kcal, the prevalence of depressive symptoms decreased [42]. A Japanese study found that carotenoids were related to depression in women and overweight elderly people, but not to men or underweight participants [34]. Conversely, a study on college students found that carotenoid intake was effective in reducing depression risk in men in a sex-stratified subgroup analysis, but not in women [41]. Table 1 and Table 2 list the detailed information and the main outcome parameters, respectively.

**Table 1:** Summary of the 13 included studies in this review with carotenoids in depression.

References	Country	Age	Gender (M/F)		Participants	Study Design	Sample Size	Types of Carotenoids	Criteria for Diagnosing Depressive Symptoms	Results	Quality Score
			Patients	Control							
May A.Beydoun [32], 2013	American	20–85	75/120	675/928	Adults	Cross-sectional study	Total: n = 1798 Patients: n = 195 Control: n = 1603	Total carotenoids	PHQ ^1^	Negative correlation (*p* < 0.001).	13
Yuri Milaneschi [1], 2012	American	≥65			Elderly	Follow-up study	Total: n = 528 Patients: n = 78 Control: n = 450	Total carotenoids	CES-D ^2^	Negative correlation (*p* = 0.04).	13
Tae-Hee Kim [33], 2015	Korea	12–18	0/35	0/245	Adolescent girls	Case–control study	Total: n = 849 Patients: n = 35 Control: n = 245	Beta– carotene	K-BDI ^3^	Negative correlation (*p* = 0.044).	14
Thao Thi Thu Nguyen [34], 2017	Japan	≥65	192/245	720/914	Elderly	Cross-sectional study	Total: n = 1634 Patients: n = 437 Control: n = 1197	Beta-carotene equivalent	GDS ^4^	Negative correlation (*p* = 0.005).	12
Xiaomin Huang [35], 2018	China	≥20	69/101	1374/1247	Adults	Cross-sectional study	Total: n = 2791 Patients: n = 170 Control: n = 2621	alpha-carotene; trans-beta-carotene; beta-cryptoxanthin; total (cis-and trans-) lycopene; lutein and zeaxanthin	PHQ-9	alpha-Carotene: no correlation (*p* = 0.62); trans-beta-carotene: negative correlation (0.02); beta-cryptoxanthin: no correlation (*p* = 0.78); total (cis-and trans-): no correlation (*p* = 0.89); lutein and zeaxanthin: no correlation (*p* = 0.09)	13
Shirin Amini [36], 2019	Iran	18–45	0/81	0/82	Postpartum women	Case–control study	Total: n = 163 Patients: n = 81 Control: n = 83	Lutein; beta-cryptoxanthin	DSM-IV ^5^	Lutein: negative correlation (*p* < 0.001); beta-cryptoxanthin: negative correlation (0.006).	14
Li Di [37], 2019	China	42–52	0/740	0/2022	Late middle-aged women	Cross-sectional study	Total: n = 2762 Patients: n = 740 Control: n = 2022	alpha-carotene; beta-carotene	CES-D	Total dietary alpha-carotene: negative correlation (0.002); total dietary beta-carotene: negative correlation (0.012).	12
Hossei [38], 2020	Iran	15–18	0/115	0/148	Female adolescents	Case–control study	Total: n = 263 Patients: n = 115 Control: n = 148	beta-Carotene	DASS-21 ^6^	beta-Carotene: negative correlation (0.036).	13
Honghan Ge [39], 2020	China	0–80	553/992	8002/7854	Adults	Cross-sectional study	Total: n = 17,401 Patients: n = 1545 Control: n = 15,856	alpha-Carotene; beta-carotene; beta-cryptoxanthin; lutein and zeaxanthin; lycopene; total carotenoids	PHQ-9	alpha-Carotene: negative correlation (*p* < 0.05); beta-carotene: negative correlation (*p* < 0.01); beta-cryptoxanthin: negative correlation (*p* < 0.05) Lutein and zeaxanthin; negative correlation (*p* < 0.01); lycopene: negative correlation (*p* < 0.05); total carotenoid: negative correlation (*p* < 0.05)	11
Sayyed Saeid [40], 2020	Iran	12–18	0/255	0/733	Adolescent girls	Cross-sectional study	Total: n = 988 Patients: n = 255 Control: n = 733	beta-carotene; alpha-carotene; lutein	21-item Beck Depression Inventory	beta–carotene: negative correlation (*p* = 0.003); alpha-carotene: negative correlation (*p* = 0.004); lutein: negative correlation (*p* = 0.031).	12
Behnoosh Boozari [41], 2021	Iran	18–43			Healthy college students	Cross-sectional study	Total: n = 184 Patients: n = 93 Control: n = 91	Total carotenoids	DASS-21	Carotenoid: negative correlation (*p* = 0.001)	12
Song Lin [42], 2021	China	≥18	126/203	1875/1901	Adults	Cross-sectional study	Total: n = 4105 Patients: n = 329 Control: n = 3776	alpha-carotene; beta-carotene; beta-cryptoxanthin; lutein/zeaxanthin; lycopene	PHQ-9	alpha-carotene: no correlation (*p* = 0.61); beta-carotene: no correlation (*p* = 0.465). Carotenoids: negative correlation (*p* < 0.001); lycopene: no correlation (*p* = 0.649); lutein/zeaxanthin: no correlation (*p* = 0.099)	12

^1^ PHQ, Patients Health Questionnaire; ^2^ CES-D, Center for Epidemiological Studies—Depression; ^3^ K-BDI, Korean version of Beck Depression Inventory; ^4^ GDS, Geriatric Depression Scale; ^5^ DSM-IV, Diagnostic and Statistical Manual of Mental Disorders; ^6^ DASS-21, Depression Anxiety and Stress Scale.

#### 3.2.1. Total Carotenoids

**Figure 2:**
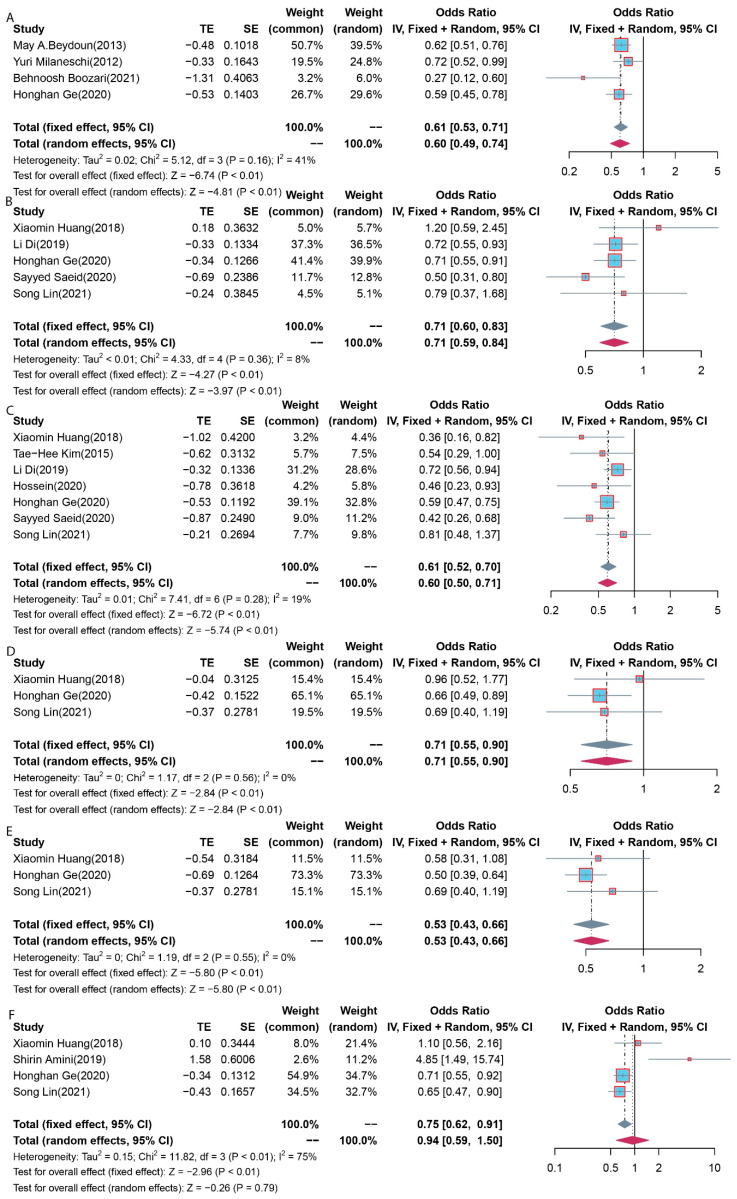
Forest plot of dietary carotenoid intake in dietary carotenoid intake vs. control groups. (**A**) total carotenoids; (**B**) alpha-carotene; (**C**) beta-carotene; (**D**) lycopene; (**E**) lutein and/or zeaxanthin; (**F**) beta-cryptoxanthin. The size of the squares corresponds to the study-specific statistical weight from each observational study, and the diamonds indicate the odds ratio with corresponding 95% confidence intervals. Horizontal lines, 95% confidence intervals (CIs).

**Figure 3:**
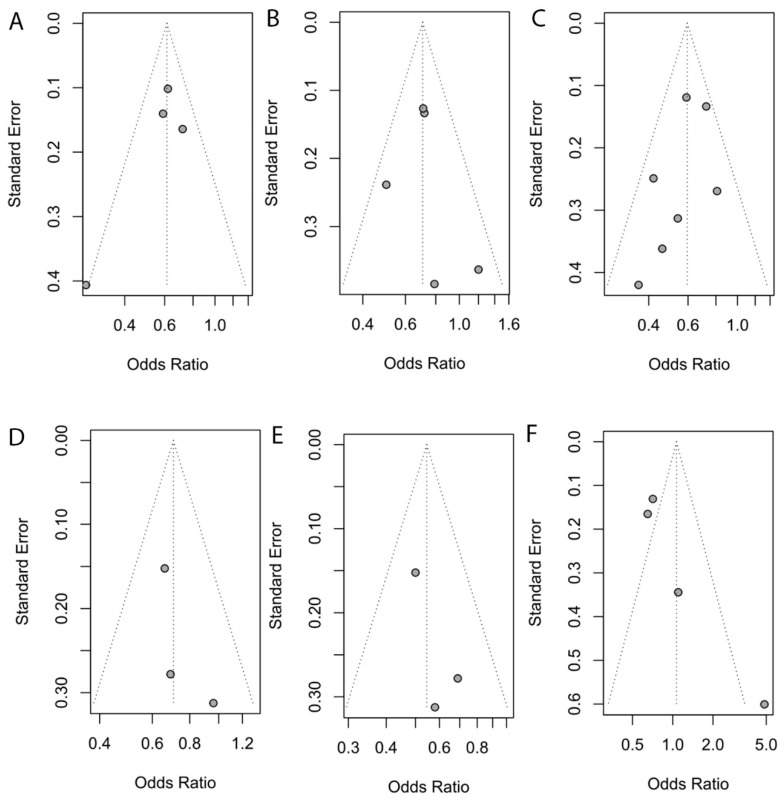
Funnel plot of the dietary carotenoid intake in dietary carotenoid intake vs. control groups. (**A**) Total carotenoids; (**B**) alpha-carotene; (**C**) beta-carotene; (**D**) lycopene; (**E**) lutein and/or zeaxanthin; (**F**) beta-cryptoxanthin.

In all four studies comprising 1632 patients with depressive symptoms and 18,199 controls with nondepressive symptoms, there were differences in the reported correlation, and all the studies described that the total carotenoid level of patients with depressive symptom was lower than that of controls with nondepressive symptoms [1,32,39,41]. In a pooled analysis, dietary intakes of the total carotenoid level were lower in the cases than those in the controls (OR: 0.61; 95% CI: 0.53–0.71; *p* < 0.01) (Figure 2A). The *I*^2^ statistic was 41%, and the *p*-value associated with the Q-statistic was 0.16, suggesting that there was no evidence for significant heterogeneity (Figure 2A). Visual examination of the funnel and Egger’s test (*p* = 0.30) show that there was no evidence of asymmetry, so there was no evidence of publication bias (Figure 3A).

#### 3.2.2. Alpha-Carotene

A total of five studies [35,37,39,40,42] with 3039 people with depressive symptoms and 25,008 without depressive symptoms as the control group reviewed the relationship between depressive symptoms and alpha-carotene. Three of these described lower alpha-carotene levels in patients with depressive symptoms compared with those in the controls without depressive symptoms [37,39,40]. Two other studies did not report significant differences [35,42]. In pooled analysis, the dietary intakes of alpha-carotene levels were lower in the cases than those in the controls (OR: 0.71; 95% CI: 0.60–0.83; *p* < 0.01) (Figure 2B). The *I*^2^ statistic was 8%, and the *p*-value associated with the Q-statistic was 0.36, suggesting that there was no evidence for significant heterogeneity (Figure 2B). Visual funnel examination and Egger’s test (*p* = 0.69) show that there was no evidence of asymmetry and thereby no evidence of publication bias (Figure 3B).

#### 3.2.3. Beta-Carotene

A total of seven studies [33,35,37,38,39,40,42] consisting of 3270 people with depressive symptoms and 25,889 without depressive symptoms as the control group looked at the relationship between depressive symptoms and beta-carotene. Six of the studies described a lower dietary intake of beta-carotenoids in patients with depressive symptoms compared with that of controls without depressive symptoms [33,35,37,38,39,40]. Only one study showed no relationship between depressive symptoms and the dietary intake of beta-carotenoids [42]. In pooled analysis, the dietary intakes of beta-carotene levels were lower in cases than those in the controls (OR: 0.61; 95% CI: 0.52–0.70; *p* < 0.01) (Figure 2C). The *I*^2^ statistic was 19%, and the *p*-value associated with the Q-statistic was 0.28, suggesting that there was no evidence for significant heterogeneity (Figure 2C). Visual funnel examination and Egger’s test (*p* = 0.23) show that there was no evidence of asymmetry, so there was no evidence of publication bias (Figure 3C).

#### 3.2.4. Lycopene

A total of three studies [35,39,42] comprising 2044 patients with depressive symptoms and 22,253 without depressive symptoms as the control group observed the relationship between depressive symptoms and lycopene. One of these studies described a lower dietary intake of lycopene in patients with depressive symptoms compared with that of the controls without depressive symptoms [39]. No significant difference was reported across two additional studies [35,42]. In pooled analysis, the dietary intakes of lycopene levels were lower in the cases than those in the controls (OR: 0.71; 95% CI: 0.55–0.90; *p* < 0.01) (Figure 2D). The *I*^2^ statistic was 0%, and the *p*-value associated with the Q-statistic was 0.56, suggesting that there was no evidence for significant heterogeneity (Figure 2D). Visual funnel examination and Egger’s test (*p* = 0.43) show that there was no evidence of asymmetry, so there was no evidence of publication bias (Figure 3D).

#### 3.2.5. Lutein and/or Zeaxanthin

A total of three studies [35,39,42] with 2044 patients with depressive symptoms and 22,253 without depressive symptoms as the control group looked at the relationship between depressive symptoms and lutein and/or zeaxanthin. One of these studies described a lower dietary intake of lutein and/or zeaxanthin in patients with depressive symptoms compared with that in the controls without depressive symptoms [39]. Two other studies did not report significant differences [35,42]. In pooled analysis, the dietary intakes of lutein and/or zeaxanthin levels were lower in the cases than those in the controls (OR: 0.53; 95% CI: 0.43–0.66; *p* < 0.01) (Figure 2E). The *I*^2^ statistic was 0, and the *p*-value associated with the Q-statistic was 0.55, suggesting that there was no evidence for significant heterogeneity (Figure 2E). Visual funnel examination and Egger’s test (*p* = 0.33) show that there was no evidence of asymmetry, so there was no evidence of publication bias (Figure 3E).

#### 3.2.6. Beta-Cryptoxanthin

A total of four studies [35,36,39,42] comprising 2125 patients with depressive symptoms and 22,036 without depressive symptoms as the control group looked at the relationship between depressive symptoms and beta-cryptoxanthin. Two of the studies described a lower dietary intake of beta-cryptoxanthin in patients with depressive symptoms compared with that in the controls without depressive symptoms [35,36]. Two other studies did not report significant differences [39,42]. In pooled analysis, there was no significant difference in the dietary intakes of lycopene (OR: 1.07; 95% Cl: 0.52–2.21; *p* < 86) (Figure 2F). The *I*^2^ statistic was 75%, and *p* < 0.01, suggesting that there was evidence for significant heterogeneity (Figure 2F). In sensitivity analysis, the OR was no longer significant following the omission of studies by Shirin Amini et al. [36]. Visual funnel examination and Egger’s test (*p* = 0.08) show that there was no evidence of asymmetry, so there was no evidence of publication bias (Figure 3F).

## 4. Discussion

In the past few decades, due to the increasing global incidence of depression, significant research has been performed on its pathogenesis. Although there is no consensus on the etiology of depression, it involves a variety of factors such as oxidative stress, and environmental, genetic, sociocultural, social, and psychological factors [43]. Oxidative damage caused by free radicals plays a significant role in the pathogenesis of depression and related mental diseases [16,44]. Therefore, in recent years, to reduce the risk of depressive symptoms, a large number of studies on antioxidants and depressive symptoms have been conducted. As important antioxidants, carotenoids have been widely used in these studies. Several studies reported no apparent relationship between carotenoid intake and the incidence of depressive symptoms [34,39]. Although most studies found that carotenoids are protective factors, the intake of carotenoids in the diet is inversely proportional to the symptoms of depression [1,32,33,35,36,37,38,40]. Like these studies, our meta-analysis shows that a high intake of carotenoids is related to and is a protective factor from depressive symptoms. In recent years, increasing evidence has shown that carotenoids play a significant role in depressive symptoms due to their antioxidant activity and scavenging effect, which gibe carotenoids great potential in treating depression disorders [40].

To the best of our knowledge, this is the first study to systematically and quantitatively assess the relationship between carotenoid intake and depressive symptoms. There is no conclusive evidence that carotenoid intake from the diet can alleviate the symptoms of depression. Our meta-analysis summarizes the results of 12 observational studies to assess the correlation between the six main carotenoids and the risk of depression. The pooled results show that depressive symptoms are significantly associated with dietary total carotenoids, alpha-carotene, beta-carotene, lycopene, lutein, and zeaxanthin intake, while this was not the case for dietary intake of beta-cryptoxanthin. The funnel plot of these results further shows that the meta-analysis is free from publication bias, as small studies reported large effects. Sensitivity analysis shows that the comprehensive results are reliable and stable.

Our results suggest that the dietary intake of carotenoids is effective in improving depressive symptoms; this is not only limited to carotenoids (total carotene, alpha-carotene, beta-carotene, lycopene, and zeaxanthin), but also to their lutein subclasses, which may have clinical significance [45]. Dietary carotenoids vary in depletion due to characteristics such as seroprevalence, water solubility, and antioxidant capacity. The most common exogenous antioxidants in plasma are carotenes: lycopene and beta-carotene [46]. Carotenoid subcarotenoids are characterized by high lipid solubility, and carotenoids are preferentially eluted and cleared under conditions of oxidative stress. In contrast, lutein is more hydrophilic, so it is consumed more slowly in plasma and tends to remain for longer [46,47]. The major carotenoids of lutein and zeaxanthin in the brain and macular region of the eye are 500-fold higher than those in plasma, and provide neuroprotection through a variety of mechanisms such as the control of free-radical-mediated damage and the scavenging of singlet oxygen. In depressive symptoms, the brain under oxidative stress may preferentially use carotenoids from large amounts of nerve tissue reserves to maintain the steady state of peripheral circulation. This theory is supported by an animal study in which zeaxanthin treatment reduced the levels of IL-6, IL-1β, and TNF-α in the hippocampus [48]. In addition, in animal experiments, lycopene (60 mg/kg) reduced lipopolysaccharide (LPS)-induced interleukin-1β (IL-1β) and heme oxygenase-1 (HO-1) levels in the plasma, and decreased the plasma levels of interleukin-6 (IL-6) and tumor necrosis factor-α (TNF-α) [49]. A cross-sectional study by Niu and his colleagues showed that a diet rich in tomatoes was related to a lower prevalence of depression symptoms in the elderly in Japan (≥70 years old), which means that the intake of lycopene in the diet may prevent the occurrence of depression symptoms [50]. Similarly, in the National Health and Nutrition Examination Survey of the United States, the intake of total carotenoids and all carotenoid subgroups was negatively correlated with depression symptoms [39]. These results suggest that carotenoids have potential therapeutic effects [51].

The relationship between beta-cryptoxanthin and depressive symptoms is largely unknown, and less research has been performed on this topic. Although all carotenoids may protect the neural tissue from depression through their antioxidant capacity [15], our study shows that the protective effect of carotenoids does not include beta-cryptoxanthin, but shows a potential trend in two other polar luteins (lutein and zeaxanthin) that are negatively associated with the prevalence of depressive symptoms. Both are polar luteins, but the reasons behind the differences in the conclusions are not clear. Bhosale put forward some reasonable reasons for the conclusions: this may be due to the mechanism of selective deposition of lutein related to specific lutein-binding proteins [52]. Another possible reason is that the nutritional and physiological characteristics of beta-cryptoxanthin may interact with exogenous substances or endogenous molecular networks in food [53,54]. To further explore the complex mechanism of cryptoxanthin, rigorous prospective cohort studies and molecular biology studies are recommended [42].

However, our study has limitations, and our findings should be interpreted with caution. First, the studies that we included were all observational (cohort, case–control, and cross-sectional studies), so directional causality could not be determined because depression may lead to reduced food intake, which reduces carotenoid intake. In addition, information such as the subject’s family history, and exercise and social-network levels that may have influenced the findings was lacking. Second, most of the studies that we included used self-reported dietary assessments that were subject to random and systematic measurement errors, and unavoidable recall bias. Third, although the included studies were adjusted for potential confounding variables, there were still other unknown confounding factors that could not be assessed, such as clinical parameters and genetic background, and the use of other antioxidants could not be excluded. Lastly, the included studies were few and all observational. There is no clinical study investigating the effect of carotenoids on depressive symptoms, because our study is only preliminary, and a large number of experimental studies are needed for further confirmation. In addition, due to the small number of the included studies, subgroup analysis was affected, and the effect of lutein and zeaxanthin on depressive symptoms could not be analyzed separately. Overall, our study shows that total intake of alpha-carotene, beta-carotene, lycopene, lutein and zeaxanthin, and carotenoids is inversely associated with the risk of depressive symptoms in adults.

## 5. Conclusions

In conclusion, the results of the meta-analysis showed that the dietary intake of alpha-carotene, beta-carotene, lycopene, lutein, and zeaxanthin, and total carotenoid intake help in reducing the risk of depressive symptoms, but the levels of beta-cryptoxanthin did not reach statistical significance. Considering the included observational studies, further longitudinal studies or clinical trials should be conducted to determine the daily intake of carotenoids and optimal plasma/serum levels to prevent or treat depression.

## Figures and Tables

**Table 2:** Summary measures for the meta-analyzed data for the six investigated carotenes.

Carotene Species	Included Studies	Patients/Control	Odds Ratio; 95% CI	*p*
Alpha-carotene	5	3039/25,008	0.71; 0.60–0.83	<0.01
Beta-carotene	7	3270/25,889	0.61; 0.52–0.70	<0.01
Total carotenoids	4	1632/18,199	0.61; 0.53–0.71	<0.01
Lycopene	3	2044/22,253	0.71; 0.55–0.90	<0.01
Beta-cryptoxanthin	4	2125/22,036	1.07; 0.52–2.21	0.86
Lutein and/or zeaxanthin	3	2044/22,253	0.53; 0.43–0.66	<0.01

## Data Availability

Data is contained within the article.
